# Stimulating the Growth, Anabolism, Antioxidants, and Yield of Rice Plants Grown under Salt Stress by Combined Application of Bacterial Inoculants and Nano-Silicon

**DOI:** 10.3390/plants11243431

**Published:** 2022-12-08

**Authors:** Khadiga Alharbi, Hany S. Osman, Emadeldeen Rashwan, Emad M. Hafez, Alaa El-Dein Omara

**Affiliations:** 1 Department of Biology, College of Science, Princess Nourah bint Abdulrahman University, Riyadh 11671, Saudi Arabia; 2 Department of Agricultural Botany, Faculty of Agriculture, Ain Shams University, Hadayek Shubra, Cairo 11241, Egypt; 3 Agronomy Department, Faculty of Agriculture, Tanta University, Tanta 31527, Egypt; 4 Department of Agronomy, Faculty of Agriculture, Kafrelsheikh University, Kafr El-Sheikh 33516, Egypt; 5 Department of Microbiology, Soils, Water Environment Research Institute, Agricultural Research Center, Giza 12112, Egypt

**Keywords:** oxidative stress, rice varieties, silica nanoparticles, plant growth-promoting rhizobacteria, soil salinity, antioxidant enzymes activity

## Abstract

The growth and development of rice face many issues, including its exposure to high soil salinity. This issue can be alleviated using new approaches to overwhelm the factors that restrict rice productivity. The objective of our investigation was the usage of the rhizobacteria (Pseudomonas koreensis and Bacillus coagulans) as plant growth-promoting rhizobacteria (PGPRs) and nano-silicon, which could be a positive technology to cope with the problems raised by soil salinity in addition to improvement the morpho-physiological properties, and productivity of two rice varieties (i.e., Giza 177 as salt-sensitive and Giza 179 as salt-tolerant). The findings stated that the application of combined PGPRs and nano-Si resulted in the highest soil enzymes activity (dehydrogenase and urease), root length, leaf area index, photosynthesis pigments, K^+^ ions, relative water content (RWC), and stomatal conductance (gs) while resulted in the reduction of Na^+^, electrolyte leakage (EL), and proline content. All these improvements are due to increased antioxidant enzymes activity such as catalase (CAT), superoxide dismutase (SOD), and peroxidase (POD), which decreased hydrogen peroxide (H_2_O_2_) and malondialdehyde (MDA) under soil salinity in rice plants compared to the other treatments. Combined application of PGPRs and nano-Si to Giza 177 significantly surpassed Giza 179, which was neither treated with PGPR nor nano-Si in the main yield components (number of grains/panicles, 1000 grain weight, and grain yield as well as nutrient uptake. In conclusion, both PGPRs and nano-Si had stimulating effects that mitigated the salinity-deleterious effects and encouraged plant growth, and, therefore, enhanced the grain yield.

## 1. Introduction

Soil salinization, which is the consequence of climate change, is one of the main restrictions to plant development and crop yield due to high levels of dissolved salts and exchangeable sodium (Na^+^) and chlorine (Cl^−^) that cause oxidative stress and damage to crop plants [1]. It is stated that more than 60% of arable lands will be menaced by 2050 as a consequence of owing to climate change, inadequate irrigation water practices, insufficient drainage systems, and chemical fertilizers. Further than 800 million ha of land is currently influenced by soil salinity [2]. Salinity stress causes oxidative damage and nutritional discharges for glycophytes. Under salt-affected soil, seed germination, seedling growth, plant development, water and nutrients absorption, physiological and biochemical properties, and grain yield can be negatively impacted [3]. In the arid and semi-arid zone, the enhancement and exploitation of saline soils for crop production has turned out to be a national approach to preserve food security [4]. In salt-affected soil, rice (Oryza sativa L as a glycophyte crop cannot grow easily or attain rational production owing to salinity stress. Rice is considered the second most important cereal crop for humans due to its nourishment, and it is more vulnerable to rhizosphere salinity compared to many other cereal crops [5]. High Na^+^ and Cl^−^ contents in salt-affected soil cause antagonistic impacts on the uptake of necessary nutrients such as K, P, Zn, Fe, Ca, Mn, and Mg ions [6,7]. Therefore, seedling growth is impeded, the number of tillers, panicles, and spikelets declines, and grain yield declines extremely. Recently, more attention has been paid to breeding salt-tolerant rice genotypes that resulted in a reasonable improvement. Nevertheless, there is not sufficient interest directed at discovering salt-tolerant genotype techniques [8]. Therefore, higher grain yield requires rigorous farming approaches to deliver sufficient and balanced amounts of necessary nutrients under salt-affected soil conditions [9].

Application of beneficial plant growth-promoting rhizobacteria (PGPR) to enhance soil rhizosphere and improve nutrient and water uptake can possibly enhance plant acquisition of nutrients and improve soil microbial activity, thus improving long-term soil fertility and increasing crop production [10]. Microbes have been an environmental and sustainable approach for augmenting crop yield under abiotic stress conditions [11,12]. Consequently, sustainable agriculture, as well as high crop production, is needed to diminish the menaces of hunger and to increase food security. In this respect, the application of seed inoculants (PGPRs) to enhance water and nutrient absorption and plant growth in plants is increasing, particularly under arid and semi-arid zones. Seed inoculation with bacterial strains could be an efficient practice that reduces the adverse environmental impacts of saline soils [13]. However, some PGPR genera have been investigated to augment the root growth and crop yields under salt-affected soil, such as Bacillus, Serratia, Pseudomonas, Azospirillum, Azotobacter, Klebsiella, and Enterobacter, which served as biofertilizers [14]. Phosphate-solubilization under salinity conditions has been approved by many of PGPRs, such as Pseudomonas sp. [15] and Bacillus sp. [16]. Pseudomonas koreensis has a great potential to tolerate salt stress by solubilizing both silicate and phosphate in the salt-affected soil, producing gibberellic acid and organic acids (malate, citrate, and acetate), which reflect on increasing the content of salicylic acid, reducing the content of ABA and strengthened the antioxidant system in plants [17]. Bacillus coagulans NCAIM B.01123, Bacillus circulance NCAIM B.02324, and Bacillus subtilis MF497446 could decline ethylene stress on root rhizosphere through secrete 1 aminocyclopropane-1-carboxylic acid (ACC)-deaminase [16]. The activity of ACC deaminase decreases ACC levels and prevents excessive increases in the synthesis of ethylene under various stress conditions, which alleviates the negative impact of soil salinity on root growth and plant development [18]. 

Rice accumulates silicon (Si) more than other plants, which indicating to the importance of Si in managing rice growth and development, increasing nutrient availability, decreases ions toxicity and minimizing both of biotic and abiotic stress in plants [19]. Under abiotic stress condition, Si is involved in the regulation of various plant metabolic processes, including the involvement in osmotic adjustment and the regulation of ROS by the antioxidant defense system [20]. The rigidity and strength of a cell wall are increased and the amount of transpiration from the cuticle is decreased by Si deposition in hulls, leaves, and culms, which improves the plant’s tolerance to abiotic stressors [21]. Silicon is not a very plant-mobile element. For a plant to grow healthily and productively throughout all stages of growth, a constant supply of Si would be necessary.

Nanoparticles as foliar spraying demonstrate a vital role in worldwide food production through the mitigation of the effects of abiotic stress [22]. Nanofertilizers actually act as efficient fertilizers by increasing the availability of essential elements for plants, resulting in efficient nutrient use as well as having attained great interest in the alleviation of the negative impacts of salt stress [23]. Furthermore, nanoparticles could be uptaken by plants more quickly and totally than conventional methods [19]. Thus, nanofertilizers could be effectively applied in extremely tiny concentrations to enhance crop production, including rice [24]. Silicon application could enhance normal rice growth by encouraging root growth, shoot weight, and physiological processes, as well as crop yield due to the absorption of larger amounts from silicon than other cereals by 10–20% [25]. Moreover, Si application as nanoparticles can increase antioxidant enzymes activity, leaf relative water content, decline Na^+^ uptake and augment K^+^ translocation while decreasing oxidative stress [26]. Nevertheless, the positive impact of nano-silica on the microbial population was also stated. Nano-silica, used as a nanofertilizer, enhanced the growth of different PGPR [27]. Karunakaran et al. [28] stated that spraying of nanoparticles improves the growth of PGPR and enhances the total soil bacterial population alongside increasing soil nutrients and stimulating rice seed germination and roots. Rajput et al. [29] stated that nanoparticles improved the growth of PGPRs and other positive populations of the soil that improved plant growth. Currently, nano-silica is described to hasten PGPR activities [30]. Nevertheless, a detailed examination of the mutual interaction of the PGPR and nano compounds is needed for their investigation of sustainable agriculture plans. The synergistic application of nano-silicon with PGPRs may provide better findings than applying them as an individual.

To date, the combination of rhizobacteria (Pseudomonas koreensis and Bacillus coagulans) and nano-silicon has not been investigated together to discover the potentialities of their motivating effects, which could enhance the growth and productivity of rice plants cultivated in salt-affected soil. The present study was designed to evaluate the impact of individual and combined treatments of microbial inoculants or nano-silicon on enhancing the physiological, biochemical, morphological, and yield attributes of a rice salt-sensitive (Giza 177) and salt-tolerant cultivar (Giza 179) planted in salt-affected soil.

## 2. Results

### 2.1. Activity of Soil Enzymes

In the salt-affected soil planted with rice plants in the 2020 and 2021 growing seasons, the activity of urease and dehydrogenase dropped dramatically in the sensitive cultivar (Giza 177) in comparison to the tolerant cultivar (Giza 179) (Table 1). Comparing the sensitive cultivar (Giza 177) to the tolerant cultivar (Giza 179), urease activity in the sensitive cultivar was reduced by 29%, while dehydrogenase activity decreased by 42%. 

The activity of soil urease and dehydrogenase was significantly boosted during both seasons by the application of PGPRs and nano-Si alone or in the combined application, mitigating the detrimental effects of soil salinity on the plant. The application of PGPRs was better than the nano-Si application in increasing the activity of urease and dehydrogenase in both cultivars. Application of PGPRs increased the activity of urease over its control by 50% and 46% for Giza 179 and Giza 177, respectively, whereas the application of nano-Si recorded a 33% and 32% increase over the control for the same cultivars (Table 1). The most effective application in activating the soil enzymes (urease and dehydrogenase) was the combined application (PGPRs + nano-Si) either in the case of sensitive or tolerant cultivars, which recorded a 96% and 93% increase than the control for Giza 179 and Giza 177, respectively (Table 1).

### 2.2. Physiological Characteristics

#### 2.2.1. K/Na Ratio

In the soil-affected soil, cultivating the susceptible cultivar (Giza 177) led to a rise in Na^+^ ion content and a reduce in K^+^ ion content in the leaves of rice plants, which recorded an increase in Na case by 35% and a reduction in K case by 75% in comparison with the tolerant cultivar (Giza 179) (Table 2). In this regard, the ability of cultivar Giza 179 to maintain the ionic equilibrium is more efficient than the cultivar Giza 177, which recorded a ration between K/Na = 0.35 in Giza 179 while in Giza 177 the recorded ratio was 0.1 in the second season (Table 2). Regardless the cultivar type (sensitive or tolerant), individual applications of PGPRs or Nano-Si and their combination significantly altered the level of K^+^ and Na^+^ ions in the leaves of rice plants grown in salt-affected soil. Application of nano-Si is more efficient than PGPRs in lowering the concentration of Na, which recorded a 25% decrease in Giza 179 and 17% decrease in Giza 177, whereas the decrease in Na content under the effect of PGPRs recorded a 16% decrease in Giza 179 and 11% decrease in Giza 177. The potential for interaction between the cultivars and the individual application (PGPRs or Nano-Si) has a positive reflection on the K content, which increased in Giza 179 under the effect of PGPRs and nano-Si by 40% and 65%, respectively. Whereas in Giza 177, these treatments were more efficient in increasing the K content over its control, which recorded 159% and 268% increase under the effect of PGPRs and nano-Si. The combined application (PGPRs + nano-Si) is superior than individual application (PGPRs or Nano-Si) in decreasing the Na levels in Giza 179 (by 36%) and Giza 177 (by 34%). Meanwhile the combined application increased the K levels in Giza 179 (by 101%) and Giza 177 (by 377%) (Table 2).

#### 2.2.2. Photosynthetic Pigments

**Figure 1:**
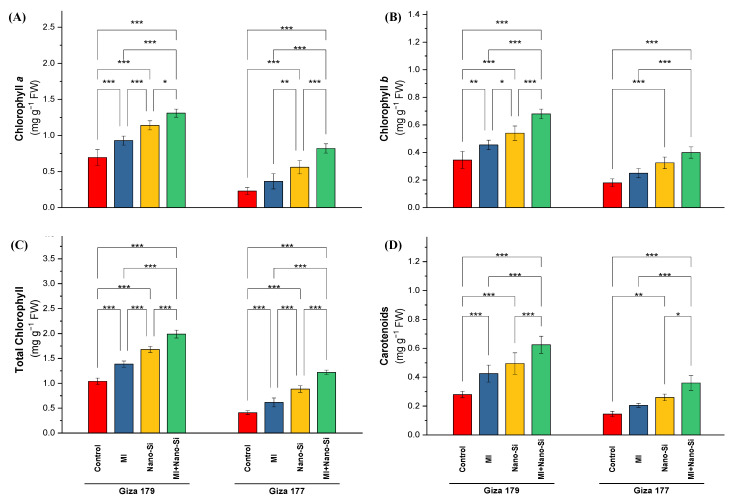
Impact of microbial inoculants (MI) and silicon nanoparticles (nano-Si) applications on the concentration of (**A**) chlorophyll a, (**B**) chlorophyll b, (**C**) total chlorophyll, and (**D**) carotenoids in the leaves of rice plants (cultivars Giza 179 and Giza 177). Data of the two seasons presented as means ± SD. *, **, and *** present the significance at *p* < 0.05, *p* < 0.01, and *p* < 0.001, respectively.

Content of chlorophylls and carotenoids as the major photosynthetic pigments in the leaves of the susceptible rice cultivar (Giza 177) were negatively affected by the cultivation in the salt-affected soil comparing to the tolerant cultivar, which recorded a reduction in chlorophyll *a*, chlorophyll *b*, total chlorophyll and carotenoids by 66%, 45%, 60% and 48%, respectively (Figure 1). Furthermore, the application of PGPRs or Nano-Si individually or combined, led to ameliorating the deleterious impacts of cultivation in salt-affected soils by consecutively increasing the photosynthetic pigments in the rice leaves. In comparison to rice plants treated with nano-Si, PGPR-inoculated plants had higher concentrations of total chlorophylls, chlorophyll *a*, chlorophyll *b*, and carotenoids in both rice cultivars. The effect of nano-Si is more significant than PGPRs application on all photosynthetic pigments, which in Giza 179 recorded an increase for chlorophyll *a*, chlorophyll *b*, total chlorophyll, and carotenoids by 49%, 72%, 56%, and 59%, respectively, while in Giza 177 (the susceptible cultivar) recorded an increase for the same pigments order by 137%, 81%, 116%, and 79% (Figure 1). In contrast to the control, the highest increase in chlorophyll *a*, chlorophyll *b*, total chlorophyll, and carotenoids was remarked with the dual application of PGPRs and nano-Si, which increased the percentage over the control by 65%, 124%, 81%, and 115% for the tolerant cultivar (Giza 179), whereas in the susceptible cultivar (Giza 177), the percentage of increments over control were 222%, 131%, 188%, and 129%, respectively (Figure 1).

#### 2.2.3. Water Relations

**Figure 2:**
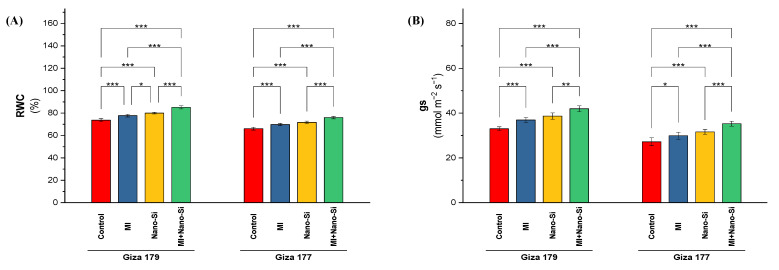
Impact of microbial inoculants (MI) and silicon nanoparticles (nano-Si) applications on (**A**) relative water content (RWC) and (**B**) stomatal conductance (gs) in the leaves of rice plants (cultivars Giza 179 and Giza 177). Data of the two seasons presented as means ± SD. *, **, and *** present the significance at *p* < 0.05, *p* < 0.01, and *p* < 0.001, respectively.

Both of the cultivars (Giza 179 and Giza 177) that were tested over the two growing seasons exhibited substantial differences in the leaves’ relative water content (RWC) and stomatal conductance (gs), particularly when PGPRs and nano-Si were used (Figure 2). Cultivating rice plants in salt-affected soil resulted in a reduction in the water relation attributes (gs and RWC) especially for susceptible cultivar (Giza 177), which reduced for both parameters by 19% and 11%, respectively than the tolerant cultivar (Giza 179). Although there is no significant differences between the individual application of PGPRs and nano-Si for both cultivars under stomatal conductance parameter and the cultivar Giza 177 under relative water content parameter, plants subjected to nano-Si exhibited a better response that subjected to PGPRs. Application of nano-Si increased the gs in Giza 179 and Giza 177 by 17% and 18%, whereas the increases in RWC were only 8% and 9% for Giza 179 and Giza 177, respectively (Figure 2). The largest increase value in stomatal conductance was reported when the PGPRs and nano-Si were applied alongside, which recorded a 26% and 32% increase for Giza 179 and Giza 177, respectively (Figure 2B). The effect of combined application on RWC was also superior of individual applications, which recorded a 15% and 16% increase for Giza 179 and Giza 177, respectively (Figure 2A).

#### 2.2.4. Oxidative Stress Indicators

**Figure 3:**
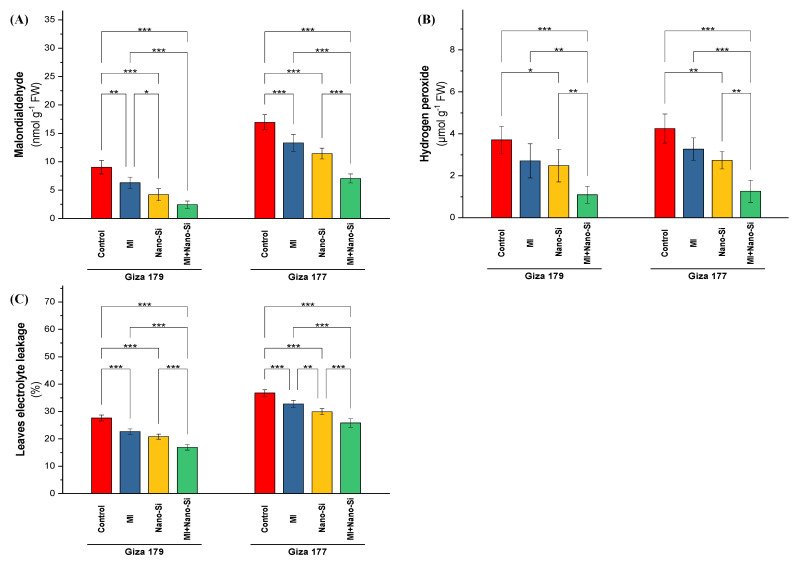
Impact of microbial inoculants (MI) and silicon nanoparticles (nano-Si) applications on the concentration of (**A**) malondialdehyde, (**B**) hydrogen peroxide, and (**C**) the percentage of electrolyte leakage in the leaves of rice plants (cultivars Giza 179 and Giza 177). Data of the two seasons presented as means ± SD. *, **, and *** present the significance at *p* < 0.05, *p* < 0.01, and *p* < 0.001, respectively.

In both seasons, cultivating rice plants in salt-affected soil enlarged values of the stress indicators (malondialdehyde (MDA), hydrogen peroxide (H_2_O_2_), and electrolyte leakage (El%)). The susceptible cultivar (Giza 177) recorded the highest values in all studied stress indicators, which reach 35% increase in EL% over its level in Giza 179. The same trend noted over values in Giza 179 with MDA (90%) and H_2_O_2_ (19%) (Figure 3). 

Values of MDA, H_2_O_2_, and EL%, in rice plants were significantly reduced under the effect of studied treatments (PGPRs, nano-Si, and combined). Nano-Si application was more significant than PGPRs application in reducing the levels of MDA, H_2_O_2_ and EL%, whereas the PGPRs significant effect was noted under MDA and EL% only (Figure 3). The ability of the tolerant cultivar (Giza179) to reducing the levels of oxidative stress indicators was better than the susceptible cultivar (Giza 177). In this regard, application of nano-Si reduced the levels of MDA, H_2_O_2_, and EL% by 59%, 45%, and 23%, respectively, in Giza 179. In contrast, in Giza 177, application of nano-Si reduced the levels of MDA, H_2_O_2_, and EL% by 32%, 33%, and 19%, respectively. 

The co-application of PGPRs and nano-Si was identified to be the most effective at reducing stress markers in rice plants. Regarding Giza 179, the application of PGPRs + nano-Si reduced MDA, H_2_O_2_, and EL% by 78%, 67%, and 39%, whereas the reduction percentage under the combined application in Giza 177 for the same parameters were 56%, 65%, and 31%, respectively (Figure 3).

#### 2.2.5. Antioxidant Defense System

**Figure 4:**
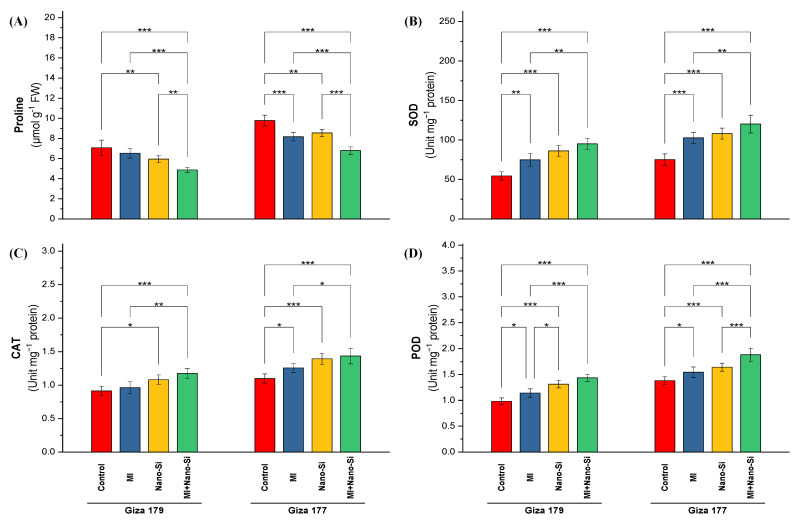
Impact of microbial inoculants (MI) and silicon nanoparticles (nano-Si) applications on (**A**) proline concentration, and the activity of (**B**) superoxide dismutase (SOD), (**C**) Catalase (CAT), and (**D**) peroxidase (POD) in the leaves of rice plants (cultivars Giza 179 and Giza 177). Data of the two seasons presented as means ± SD. *, **, and *** present the significance at *p* < 0.05, *p* < 0.01, and *p* < 0.001, respectively.

The amount of proline, a non-enzymatic antioxidant, reached its peak concentration in the leaves of rice-sensitive cultivar (Giza 177) growing in salt-affected soil comparing to its amount in Giza 179 (Figure 4A). Proline levels in the tolerant cultivar (Giza 179) reduced by 25% than its level in Giza 177. Under Giza 177, application of PGPRs is more pronounce than nano-Si application in reducing the proline concentration, which was able to minimize its level by 19% comparing to 12% for nano-Si. In contrast, under Giza 179, application of nano-Si was more efficient than PGPRs, which reduced the proline concentration by 17% comparing to 8% reported by PGPRs application (Figure 4A). The lowest levels in proline concentration in both cultivars (Giza 179 and Giza 177) were recorded from the combined treatment (PGPRs + nano-Si), which reached 32% and 30% reduction percentage in both cultivars, respectively (Figure 4A).

Activities of the antioxidant enzymes such as superoxide dismutase (SOD), catalase (CAT), and peroxidase (POD) in the leaves of rice plants growing in salt-affected soil are higher in the susceptible cultivar (Giza 177) than the tolerant cultivar (Giza 179) (Figure 4). The percentage of increase in the activity of SOD, CAT, and POD recorded 42%, 23%, and 43%, respectively. Application of PGPRs and nano-Si individually or combined were more efficient in increasing the activity levels comparing to its control in Giza 179. In the time of recordings 36% and 16% increases in SOD and POD activity under PGPRs application, the application of nano-Si recorded 61% and 34% increase over the control. Catalase activity was more significant in Giza 177 than Giza 179. Application of PGPRs and nano-Si increased the catalase activity by 15% and 27%, respectively, whereas the increases in Giza 179 for same treatments were 5% and 20%, respectively (Figure 4). 

Combined application (PGPRs + nano-Si) recorded the maximum enzyme activity in both rice cultivars. The percentage of increase in Giza 179 for the activities of SOD, CAT, and POD recorded 79%, 31%, and 48%, respectively, while the increase recorded in Giza 177 for the same enzymes were 63%, 33%, and 35%, respectively (Figure 4).

#### 2.2.6. Vegetative Characteristics

**Figure 5:**
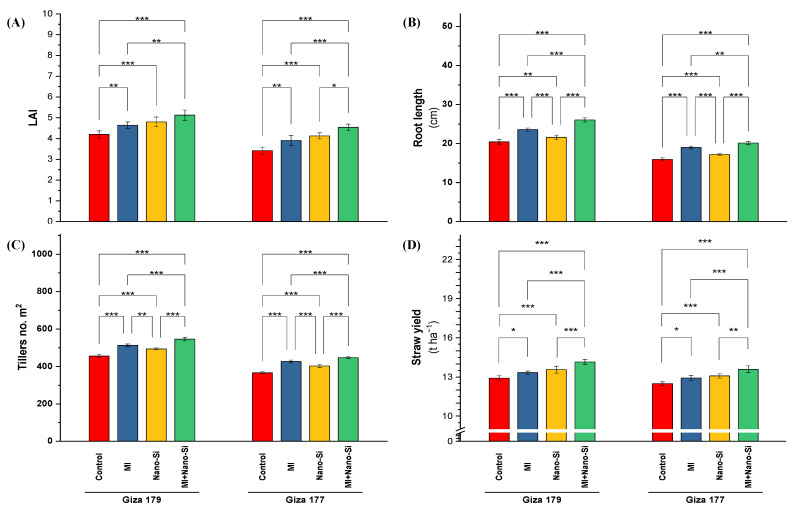
Impact of microbial inoculants (MI) and silicon nanoparticles (nano-Si) applications on (**A**) leaf area index (LAI), (**B**) root length, (**C**) number of tillers m^−1^, and (**D**) straw yield of rice plants (cultivars Giza 179 and Giza 177). Data of the two seasons presented as means ± SD. *, **, and *** present the significance at *p* < 0.05, *p* < 0.01, and *p* < 0.001, respectively.

Leaf area index (LAI) is most visible indicator reflecting the effect of abiotic stress on plant growth. In this regard, the susceptible rice cultivar (Giza 177) recorded a 19% reduction in LAI comparing with the cultivar Giza 179 (Figure 5A). Application of nano-Si is superior to PGPRs in increasing LAI, which recorded under Giza 179 and Giza 177 percentage of increase equal 13% and 22%, respectively. Combined application maximizing the LAI in both cultivars, which recorded 20% and 34%, respectively (Figure 5A).

Root length of rice plants cultivated in salt-affected soli is another growth parameter exhibited sensitivity to salinity stress by reducing its value in Giza 177 by 22% than Giza 179 (Figure 5B). Plant growth-promoting rhizobacteria is more significant than nano-Si application in increasing the root length in both cultivars (Giza 179 and Giza 177), which recorded 16% and 19% percentage of increase, respectively. In contrast, nano-Si recorded only 5% and 9% percentage of increase, respectively for both cultivars. The best significant effect recorded with the combined application (PGPRs + nano-Si), which increased by 29% and 27% for both cultivars, respectively (Figure 5B).

Number of tillers in the rice cultivar Giza 177 recorded a reduction reach of 20% below, which recorded with the tolerant rice cultivar (Giza 179) (Figure 5C). Application of PGPRs is more effective than nano-Si in increasing the tillers number, which increased its levels by 13% and 16% for Giza 179 and Giza 177, respectively, comparing to 9% and 10% for nano-Si. Application of combined application in both cultivars increased the tillers number about one-fifth over control, which recorded 20% and 23% increase over control for both cultivars (Figure 5C).

Rice straw yield represents the outcome of vegetative growth under growing conditions. The overall reduction in the straw yield of the susceptible rice cultivar (Giza 177) compared to the tolerant cultivar (Giza 179) was only 3.6% (Figure 5D). Application of nano-Si is more effective than PGPRs in increasing the straw yield, which recorded a 3.8% and 4.7% increase for Giza 179 and Giza 177, respectively. The highest straw yield in both cultivars was noted with the mixed application (PGPRs + nano-Si), which recorded a 9.1% and 8.6% increase over the control of each cultivar, respectively (Figure 5D).

#### 2.2.7. Reproductive Growth Characteristics

**Figure 6:**
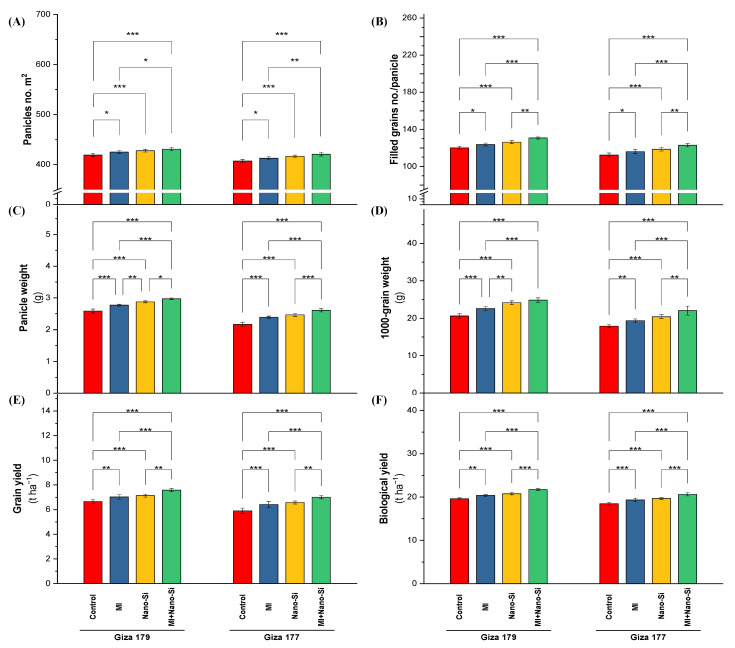
Impact of microbial inoculants (MI) and silicon nanoparticles (nano-Si) applications on (**A**) number of panicles m^−1^, (**B**) number of filled grains panicle^−1^, (**C**) panicle weight, (**D**) 1000-grain weight, (**E**) grain yield, and (**F**) biological yield of rice plants (cultivars Giza 179 and Giza 177). Data of the two seasons presented as means ± SD. *, **, and *** present the significance at *p* < 0.05, *p* < 0.01, and *p* < 0.001, respectively.

The susceptible cultivar of rice plants (Giza 177) growing in salt-affected soil experienced negative effects and significantly decreased levels of the yield-related parameters (panicles no. m^2^, number of filled grains planicle^−1^, panicle weight, 1000-grain weight, and grain yield) (Figure 6). The percentage of reduction in Giza 177 in comparison to Giza 179 for panicles no. m^2^, number of filled grains panicle^−1^, panicle weight, 1000-grain weight, and grain yield were 3%, 7%, 17%, 13%, and 13%, respectively.

Individual treatments of PGPRs or Nano-Si, in addition to their combinations under all investigated cultivars resulted in a positive improvement in the yield-related characteristics. Application of PGPRs recorded increases in the panicles no. m^2^ in Giza 179 cultivar by 1.6% over control, number of filled grains planicle^−1^ by 3%, panicle weight by 8%, 1000-grain weight by 10%, grain yield by 6%, and biological yield by 4% (Figure 6). Regardless the increases in yield attributes under PGPRs, application of nano-Si recorded a better effect on these parameters, which leading to increases in the panicles no. m^2^ in Giza 179 cultivar by 2% over control, number of filled grains panicle^−1^ by 6%, panicle weight by 12%, 1000-grain weight by 19%, grain yield by 7%, and biological yield by 5%. 

The co-application of PGPRs + nano-Si maximized the panicles no. m^2^, number of filled grains panicle, panicle weight, 1000-grain weight, grain yield, and biological yield regardless of the cultivar type, which recorded increases these parameters over its control in Giza 179 by 3%, 9%, 17%, 21%, 15%, and 11%, respectively, while the increases for the same order of parameters in Giza 177 were 3%, 10%, 22%, 21%, 21%, and 12%, respectively (Figure 6).

#### 2.2.8. Content of N, P, K, Na, and Si in Rice Grains

The capability of rice grains to uptake N, P, K, and Si was considerably lessened in the susceptible cultivar growing in salt-affected soil during both growing seasons comparing to the tolerant cultivar, which reduced the nutrients content of N, P, K, and Si by 10%, 63%, 43%, and 34%, respectively while the Na content in rice grains recorded a 79.6% in susceptible cultivar (Giza 177) over the tolerant cultivar (Giza 179) (Table 3). 

Although there are no significant differences between the individual application either with PGPRs or Nano-Si on the grains content of N, both of them increased the N content when compared with its control by 12% and 14% in Giza 179, and by 7% and 12% in Giza 177 (Table 3). In contrast, application of nano-Si was superior to PGPRs on increasing the grains content of P, K, and Si in Giza 179 by 86%, 105%, and 55% over its control. Meanwhile the Na content decreased under the treatment of nano-Si by 46% in Giza 179 and by 25% in Giza 177.

When rice plants were treated with the co-application of PGPRs + nano-Si during both growing seasons, significant increases in the grain content of N, P, K, and Si were shown, which counteracted the negative effects of salt stress (Table 3). Regardless of cultivar type, it was discovered that rice plants treated with a combined application of PGPRs and nano-Si absorbed more N, P, K, and Si from the soil over the course of two years, which increased the grain content in Giza 179 from N, P, K, and Si by 17%, 144%, 160%, and 73%, respectively, whereas the Na content decreased to its lowest level, which reduced by 56% (Table 3).

## 3. Discussion

Salt-affected soil is a damaging ecological problem that is deemed as one of the key reasons of decrease in sustainable agricultural production resulting in dysfunction, which occurs in plant physiological and biochemical processes along with the antioxidant resistances due to the extreme production of reactive oxygen species (ROS) [31]. Furthermore, salt-affected soil causes the decrease in cell membrane stability and lipid peroxidation owing to the boosted sodium ions accompanied by augmented reactive oxygen species [32]. Salt-affected soil causes ROS production in cellular organelles such as chloroplasts, peroxisomes, and mitochondria, that can damagingly influence various physiological and biochemical processes such as relative water content, photosynthesis, stomatal conductance, antioxidant enzymes activity as well as plant development [33]. Nanoparticles as foliar spraying and bacterial inoculants as seed inoculation have newly obtained its pathway into agricultural systems, which have an important possibility for enhancing of crop production and alleviating the negative impacts of the abiotic stress [34]. Our examinations have investigated that the exploitation of nanoparticles as foliar spraying and bacterial inoculants as seed inoculation resulting in augmented the efficiency of the nutrient elements under salt-affected soil. Moreover, an easy translocation and ensure nutrients delivery to different systemic organs such as leaves, roots, and stems. While the individual characters of plant growth-promoting rhizobacteria and silicon nanoparticles are well determined in saline alleviation, nevertheless, their coupled addition had not been investigated [35]. Consequently, in this investigation, the coupled application of nano-Si and two bacterial strains inoculation was assessed. Root growth is one of the extremely susceptible and imperative stages that are harshly influenced by salinity. Salt stress causes non-uniform germination and immature roots especially owing to restrained nutrient and water uptake [36]. 

Salt stress constrains the soil enzyme activity (urease and dehydrogenase) and interrupts morphological and physiological characteristics [37]. The amelioration in root growth could be owing to the promotion of growth hormones that augmented the soil enzymes activity, such as urease and dehydrogenase, that in turn improved carbohydrate assimilation. The augmented levels of auxin and cytokinin that may be a significant contributor resulting in increased soil enzymes activity augment cell division and elongation [38]. Two bacterial strains inoculation could promote compatible solutes such as proline and sugars, which diminish membrane impairment that is able to osmoregulate in plant cells and sustain their physiological performance and declines stress intensity [39]. Our findings proved that two bacterial strains’ inoculation augmented root growth and soil enzyme activity levels under salt stress. In this examination, the impacts of nano-Si on rice growth under salt stress were studied. The findings showed a vital role in the root growth and leaf area expansion of rice. Nevertheless, it is outstanding to observe that nano-Si exhibited to be more effective as compared to control treatment [22]. It could be owing to the reason that nano-Si could accelerate the water transportation in plants that increase physiological characteristics [26,40]. Foliar spraying with nano-Si, it increases the antioxidant defense and decrease the oxidative damage, which results in improved morphological and physiological attributes due to activating the metabolic system under soil salinity [41,42]. 

Salt stress causes a noticeable decrease in grain yield and photosynthetic capacity of crop plants. It is owing to reactive oxygen species and oxidative damage resulting in a decline in the growth and development of crops [43]. During this investigation, it was stated that a single application of two bacterial strains and nano-Si showed a marked decline in the damaging impacts of salt stress on rice plants [44]. Their coupled addition moreover increased their positive impacts; therefore, it was implied that Nano-Si and PGPR enhanced the plant defense responses. The role of two bacterial strains inoculation in the production of auxins is named IAA. This IAA looks to improve the growth and development of crop plants by contributing to many processes such as cell elongation, meristematic tissue and the introduction of lateral roots and shoots and the accumulation of saccharides [45,46]. Cytokinins also improve the number of grains and grain yield in crops and play a vital role in meristem morphogenesis, which assists flower meristem development as well as the balance between the sources and sink [45,47]. Therefore, the findings of this study presented that coupled Nano-Si and PGPR boosted chlorophyll a, b, and carotenoids under salt-affected soil. Mechanisms that helped plant root growth and chlorophyll content may include augmenting nutrients and water uptake [48]. 

The hurtful effects of salt-affected soil decline rice growth and production due two causes, high osmotic damage and ion toxicity due to toxic ions such as Na^+^ and Cl^−^ as stated in our investigation [49]. The seed inoculation with of two bacterial strains has a great potential to reduce Na^+^ translocation due to the excretion of IAA and bacterial exopolysaccharide, which could bind Na^+^ and prevent its uptake in plants moreover augment K^+^ translocation [50]. PGPR boosted cell enlargement in the root and permitted rice roots to absorb further K^+^ and run Na^+^ ions out under soil salinity conditions [50]. Application of nano-Si as foliar spraying enhanced cell elongation and ion selectivity whereas declined the injurious effect of Na^+^ ion and enhanced plant growth under saline soil compared to control treatment [51]. 

In this study, the coupled application of PGPR and nano-Si could be a valuable technology to withstand growing under soil salinity. Furthermore, photosynthetic pigments (chlorophyll a, b, and carotenoids) and physiological attributes such as relative water content, stomatal conductance, electrolyte leakage, and proline content) have been deemed beneficial parameters under salt-affected soil [52]. The reduction in photosynthetic pigments and physiological attributes under soil salinity is mainly due to chloroplast stresses resulting in decreased plant growth and crop productivity [53]. The improvement of photosynthetic pigments (chlorophyll a, b, and carotenoids) and physiological attributes such as relative water content, stomatal conductance, electrolyte leakage, and proline content) could be attributed to PGPR application as seed inoculation in promoting meristematic activity, which causes an increase in cell division and enlargement [19]. PGPR application has the potential to water uptake together with boost nutrient uptake. Moreover, IAA-producing bacteria enhance soil health, such as the soil’s physical and chemical attributes [19]. It has been confirmed, certainly, that PGPR can improve roots and leaves due to its positive effect on osmolytes, photosynthetic pigments, and physiological parameters [52]. These findings are reliable with previous findings such as those stated by [54]. It was demonstrated that foliar-applied by nano-Si is rapidly absorbed by vacuoles, and it collects mainly in the cytosol. Hence, nano-Si application increases plant growth under soil salinity because of membrane integrity [55]. Additionally, foliar spraying with nano-Si exhibited significant potential for enhancing the biosynthesis of photosynthetic pigments, such as chlorophyll a, chlorophyll b, and carotenoids, in addition to physiological processes such as stomatal conductance and relative water content while decreasing electrolyte leakage and proline content under salt-affected soil in rice plants due to osmoregulation and influencing hydraulic conductivity [23]. Nano-Si application has a positive influence on oxidative phosphorylation, protein polymerization, and enzymatic activities [23]. It was proved in our study that synergistic application of PGPR with nano-Si had a further direct impact on osmolytes, photosynthetic pigments, and physiological attributes than an individual application under salt-affected soil that stimulated mineralization, organic acids, and increased plant nutrient availability. These results are in conformity with those recorded by Ding et al. [56]. 

In the current investigation, the antioxidant enzymatic activities such as catalase, superoxide dismutase, and peroxidase were boosted in rice plants. Nevertheless, this augment was not satisfactory in detoxifying the adverse impacts of ROS. Inversely, the individual application of two bacterial strains resulted in a clear augment in catalase, superoxide dismutase, and peroxidase activities [57]. Nonetheless, it was detected that catalase, superoxide dismutase, and peroxidase activities are further augmented under the coupled application of two bacterial strains, and nano-Si may be owing to diminished sodium uptake [44]. Catalase, superoxide dismutase, and peroxidase activities might convert H_2_O_2_ into non-toxic compounds such as (H_2_O and O_2_), thus defending the plants from their harmful impacts on cell membranes and macromolecules [58]. It was also observed higher H_2_O_2_ contents, which cause membrane humiliation and lipid peroxidation that augment oxidative stress [59]. From our findings, it was demonstrated that individual application of two bacterial strains or nano-Si under salt-affected soil caused an observed decline in H_2_O_2_ levels and reduction in lipid peroxidation (MDA) compared to untreated plots [59]. However, the synergistic application of two bacterial strains and nano-Si was further effective in alleviating oxidative damage compared to the sole application, resulting in transforming ROS into less or non-toxic compounds [60], which resulting in eventually a highly positive impact on physiological processes, plant growth, and yield-related traits under soil salinity [61]. The enhancement in plant growth could be ascribed to the biosynthesis of phytohormones such as IAA, that might mainly be connected to the yield and its traits, augmenting nutrients, the activity of 1-aminocyclopropane-1-carboxylate (ACC) deaminase, and osmolyte production [62]. In our examination, the decline in the number of panicles/m^2^, 1000 grain weight, and the number of grains/panicles resulted in adversely impacting grain yield, straw yield, and harvest index under salt-affected soil [63]. It was attributed to the reduction in nutrient and water uptake through roots and plant organs to grain filling. In addition, salt-affected soil causes impairment in the ovary, which adversely affects grain filling. It was found from our study that seed inoculation with the two bacterial strains and/or foliar spraying with nano-Si resulted in further improvement crop related-traits and production compared to untreated plots [64]. 

The synergistic application of the two bacterial strains and nano-Si alleviated the adverse effect of soil salinity, which could decline sodium uptake and increase potassium uptake as well as leaf area, which in turn reduces oxidative stress and improves photosynthesis rate, panicle fertility, and heavy panicles [60]. The yield data displayed that untreated plants were vulnerable to soil salinity, resulting in considerably less grain production. The increase in Na+ ions will stop the photosynthesis process by decreasing cell organelles, lessening enlargement and differentiation of tissues, nutritional imbalance, and triggering cell membrane injury [65]. These negative impacts of salt stress could be declined with the application of salt-tolerant two bacterial strains, as demonstrated in this study. The seed inoculation with the selected two bacterial strains has significantly augmented the photosynthesis of two rice cultivars, resulting in higher grain yield under saline soil [66]. Rice plants subjected to salt-affected soil declined due to a decrease in essential elements uptake from the soil, especially N, P, K, and Si contents, while an increase in Na^+^ ions resulted from osmotic stress [67]. Seed inoculation with two bacterial strains improved soil nutrient cyclings such as N, P, K, and Si for plant absorption while decreasing Na+ ions uptake owing to the augmented levels of auxin and cytokinin that may be a significant contributor resulting in increased soil enzymes activity [68]. Foliar-applied nano-Si could also improve nutrient cycling in leaves such as N, P, K, and Si for plant absorption while decreasing Na^+^ ions uptake. Nano-Si could also have relative water content and nutrient uptakes such as N, P, and K and prevent Na influx, which enhances leaf water content and photosynthesis. It was stated that nano-Si improved the transport of N, P, K, and Si from leaves to grains to support grain formation while decreasing Na uptake [69,70]. The further increase in N, P, K, and Si contents in grains was more observed with the synergistic application of two bacterial strains and nano-Si. Generally, our investigation showed that application of two bacterial strains and nano-Si on salt-stressed rice plants mitigates the adverse impacts of salinity by improving water status, augmenting photosynthetic rate, lessening oxidative stress, regulating some osmolytes and phytohormones along with augmenting antioxidant enzyme activity and, therefore, increasing yield production and nutrient uptake.

## 4. Materials and Methods

### 4.1. Experimental Layout and Treatments

The present examination was investigated at the Experimental Farm of the Elamaar village in the region of Sidi Salem (31°07′ N latitude, 30°57′ E longitude), Kafr El-sheik Governorate, Egypt during 2020 and 2021 summer seasons to study the application of plant growth-promoting rhizobacteria (*Pseudomonas koreensis* and *Bacillus coagulans*) and silicon in nanoparticle form (nano-Si) on the moro-physiological characteristics and yield-related traits as well as the productivity of Giza 177 as the salt-sensitive cultivar and Giza 179 as the salt-tolerant cultivar under salt-affected soil. The experimental design was split-plot arranged into randomized complete blocks with three replicates. The treatments were eight combinations including two varieties (Giza 177 and Giza 179) which were distributed in the main plots and four applications (control, PGPRs, Nano-Si and PGPRs + nano-Si) which were distributed in the sub plots. PGPRs (two bacterial strains; 950 g ha^−1)^ was applied as seed inoculation before planting in the saline clay soil. However, nano-Si (12.5 mg/L) was applied twice as foliar spraying on plant leaves at mid tillering stage and mid booting stage. Mixture of used strains (1:1) were prepared as peat-based inoculums, 15 mL of 10^8^ CFU mL^−1^ from each culture per 30 g of the sterilized carrier and spread over a plastic sheet away from direct sun for 20 min before application. Nano-Si solution was prepared at the Agricultural Microbiology Department, Soils, Water and Environment Research Institute (SWERI), Sakha Agricultural Research Station, Agriculture Research Center (ARC), Kafr El-Sheikh, Egypt. The properties of applied nano-Si (SiO_2_) were 260-320 m^2^ g^−1^ for specific surface area, 4–4.5 for pH, 10 nm for diameter. Soil samples were assembled before planting from 0 to 30 cm depth by an auger and its physicochemical analysis characteristics were analyzed based on Black et al. The soil analysis is shown in Table 4. 

After soaking the rice seeds for a night in fresh water steeped for another 24 h, the rice seeds were distributed on the nursery land at the rate of 120 kg ha^−1^. After one month, the rice plants were transplanted into the permanent land at the rate of 3–4 seedlings per hill with spacing 20 × 20 cm between hills and rows in 25 m^2^ (5.0 m × 5.0 m) size plots and repeated three times at June 3rd in 2020 and June 4th in 2021. The nursery land was fertilized with calcium super phosphate (15.5% P_2_O_5_) and was added at 125 kg P_2_O_5_ ha^−1^ pre-transplanting. Total N fertilization was applied at 160 kg N ha^−1^ as urea (46.5%) and added on three equal doses through rice growth. Plots were maintained flooded until 15 days pre-harvesting.

### 4.2. Soil Enzymes Activity

At panicle initiation stage, soil enzymes activity (urease and dehydrogenase) were measured. Urease activity was calculated using the quantification of ammonia by the Spectrophotometric technique at 660 nm as explained by Kandeler and Gerber [71]. The activity of dehydrogenase was estimated by Öhlinger and Von Mersi [72], as the soil samples were mixed with INT-solution, after maintaining of samples with sucrose (8%) for 3 h at 37 °C. The reduced triphenyltetrazolium chloride (TTC) was extorted with dimethyl-formamide and ethanol and determined photometrically at 464 nm. The model UV-160A spectrophotometer (Shimadzu, Japan) was used in these analyses.

### 4.3. Physiological Characteristics

#### 4.3.1. Leaf Na^+^ and K^+^ Determination

At panicle initiation stage, five leave samples were selected from each plot to determine Na^+^ and K^+^ contents (mg kg^−1^ DW) using ultra-pure water the volume of the sample was added to 50 mL in a volumetric flask. According to Temminghoff and Houba [73], Na^+^ and K^+^ contents were determined by AAS (Perkin Elmer 3300) with a detection limit of 100 ppb.

#### 4.3.2. Chlorophylls and Carotenoid 

Chlorophyll a, b, and carotenoids were determined at the panicle initiation stage, according to Peng and Liu [74]. Five fresh leaves were washed to remove the impurities pre-extraction. Subsequently, 2 g of the leaves were obtained and homogenized in 80% acetone by the mortar and pestle. The extracts were centrifuged. The absorbance was calculated at 663, 645, and 470 nm by a spectrophotometer. Chlorophyll a, b, and carotenoids were computed as mg g^−1^ FW.

#### 4.3.3. Water Relations

##### Relative Water Content (RWC)

Determination of RWC was determined according to the method described by Weatherley [75]. Ten topmost fully expanded leaves at the panicle initiation stage from each treatment were weighted to compute the fresh weight (FW). These leaves were then rehydrated in distilled water in 100 mL beakers and put there until the saturation point for 24 h to weighted and compute the turgid weight (TW). The same leaves were dehydrated in the oven for 48 h to obtain the dry weight (DW). RWC = [(fresh mass − dry mass)/ (saturated mass − dry mass)] × 100.

Relative water content was computed using the following equation:

$$\mathrm{RWC}\;\left(\%\right)=\left[\left(\mathrm{FW}\;-\;\mathrm{DW}\right)\;/\;\left(\mathrm{TW}\;-\;\mathrm{DW}\right)\right]\times 100$$

##### Stomatal Conductance

Stomatal conductance was determined at the panicle initiation stage by a dynamic diffusion porometer (Delta-T AP4, Delta-T Devices Ltd., Cambridge, U.K.) for five days from the abaxial and adaxial surfaces of five leaves. Measurements in the top leaf and front (ra) and back side (rb) of the center of the leaf.

Total leaf conductance (rl) is 1/rl = 1/ra + 1/rb.

#### 4.3.4. Oxidative Stress Indicators

##### Lipid Peroxidation

At the panicle initiation stage, the MDA content in leaves was estimated to calculate the membrane stress. So, thiobarbituric acid reactive substances (TBARS) were evaluated using the method obtained by Du and Bramlage [76]. About 0.5 g leaf was mixed ground in liquid N_2_ and hydro-acetone buffer (4:1 *v*/*v*).0.65% thiobarbituric acid (TBA) and 0.01% Butyl hydroxyl toluene (BHT) were added, and samples were brewed at 95 °C. After incubation, the homogenously extracted was subjected to centrifugation at 10,000 × *g* for 15 min. The homogenously extracted was estimated spectrophotometrically at 532 and 600 nm computed in nmol g^−1^ FW.

##### H_2_O_2_ Content

At the panicle initiation stage, the H_2_O_2_ content of leaves was measured colorimetrically using the method of Velikova et al. [77]. Representative five leaves (0.5 g) were homogenously extracted by liquid N_2_ and trichloroacetic acid (TCA: 0.1%). It was extracted by centrifugation of the homogenized sample at 3000 rpm for 20 min. Nonetheless, the analysis homogenized sample was determined by 10 mM K-phosphate buffer (pH 7.0, 1.0 mL), potassium iodide (2 M, 1 mL), and plant extract (1 mL), and then the mixture absorbance was recorded at 390 nm by a spectrophotometer. A standard curve was likewise designed under standard circumstances, and H_2_O_2_ contents were determined as µmol g^−1^ FW. 

##### Electrolyte Leakage

To determine the leakage of ions from membranes at the panicle initiation stage, five leaves were obtained and washed with distilled water from each treatment. They were put in test tubes, including 10 mL distilled water, and maintained in a water bath at 50 °C for 25 min, and electrical conductivity (C1) was noted. Then, the same samples were put in a water bath for 15 min at 80 °C, and electrical conductivity (C2) was recorded. The electrolyte leakage was computed according to the equation of Naghashzadeh [78]. EL = [C1/C2] × 100.

#### 4.3.5. Antioxidant System

##### Proline Content

Proline content was estimated spectrophotometerically [79]. For this, leaf samples of the rice plant (0.5 g) at the panicle initiation stage were homogenized with 4 ml of sulfosalicylic acid (3.0%) in a mortar and stored for 24 h at 5 °C. The solution was centrifuged at 3000 rpm for 5 min at room temperature. The supernatant was homogenized up with 4 mL of acidic ninhydrin reagent. The solution was automatically shaken; then, these tubes were ovened in the water bath for 60 min. After cooling and the solution was attained with 4 mL of toluene. The diffusion of the toluene layer was absorbance at 520 nm. The absorption was observed with a calibration curve and expressed as μ mol proline g^−1^ FW.

##### Antioxidant Enzymes Activity

Extracts of enzymatic antioxidants were made by freezing different leaves samples (1 g) in liquid nitrogen to avoid proteolytic activity, then grinding with 5 mL of cold extraction buffer (0.1 M phosphate buffer, pH 7, containing 0.5 mM EDTA, and 2% [*w*/*v*] PVP), and centrifuging for 20 min at 10,000 × *g* to use the supernatant as enzyme extract [80,81].

##### Superoxide Dismutase (SOD)

Superoxide Dismutase (EC 1.15.1.1) was assessed by calculating the volume of dye nitroblue tetrazolium (NBT) which was declined when superoxide radicals were produced [82] The reaction combination (containing of 100 mL SOD extract in 50 mM K-phosphate buffer, 13 mM methionine, 0.075 mM NBT, 0.10 mM EDTA, and 0.002 mM riboflavin) was subjected to light for 15 min in a light room to estimate superoxide dismutase activity. The absorbance of the blue formazan dye that was produced was calculated exactly at 550 nm relative to the absorbance of the reaction mixture that did not contain any sample. Superoxide dismutase is measured as unit mg^−1^ protein.

##### Catalase (CAT)

Catalase (EC 1.11.1.6) activity was measured by the technique obtained by Aebi [83]. The enzyme extract was made with 0.5 mL of 0.2 M H_2_O_2_ in 10 mM K-phosphate buffer (pH 7.0) before being examined. Catalase enzyme was evaluated using a spectrophotometer at 240 nm by the consumed level of H_2_O_2._ Catalase (EC 1.11.1.6) activity is measured as unit mg^−1^ protein.

##### Peroxidase (POD)

Peroxidase (EC 1.11.1.7) activity was evaluated using the method of Vetter et al. [84]. Peroxidase activity in the samples was assessed by mixing 100 μL enzyme extract with 2.9 mL 50 mM phosphate-citrate buffer (pH 6.5), 0.03% H_2_O_2_, and 0.1% ortho-phenylenediamine in the assay combination. At 430 nm, the change in absorbance was calculated for 5 min. The peroxidase activity is measured as unit mg^−1^ protein.

#### 4.3.6. Vegetative Growth Attributes

##### Leaf Area Index (LAI)

Leaf area was determined by detaching leaves from the shoot at the panicle initiation stage, washing the leaves with distilled water, and dehydrating them with tissue paper. The area of leaves was estimated by a leaf area meter (Model LI-COR, Inc., Lincoln, NE, USA). Leaf area index (LAI) was computed at 50 days after transplanting by the formula, as suggested by Evans [85].

##### Root Length

At anthesis, plant samples, including roots, were gently obtained to measure root length (cm). Root length was measured as the length of the root from the base of the plant to the tip of the main axis of the primary root.

#### 4.3.7. Reproductive Growth Attributes

##### Crop Yield

At the maturity stage, panicles of five random hills from each experimental unit were calculated and transformed to the number of panicles/m^2^. Panicles number was calculated from each hill, every panicle was hand-threshed, and the unfilled panicles were detached from filled panicles by a blower. A total of 10 panicles were selected from every plot to assess the number of grains/panicles and 1000 grain weight (g). The biological yield (both grain and straw yield ton ha^−1^) was calculated from a 6-m^2^ area in every plot, and the standard grain moisture content of 14% was applied to yield calculation [86].

##### Nutrients Content in Rice Grains

At the maturity stage, ten panicles were obtained randomly from the middle of the plots. Fifty grains were obtained from each experimental unit, rinsed with mineral water, and heated for 48 h at 70 °C to evaluate grain N, P, and K contents (g kg^−1^). The dehydrated grains were crushed with a stainless-steel mill and digested with HNO_3_-H_2_O_2_ solution (2:1). P content was calorimetrically evaluated using the technique of Sparks et al. [87]. The K content was evaluated using AAS (Perkin Elmer Ltd., UK) with a detection limit of 100 ppb (Sparks et al.). An additional 1 g powder aliquot was digested with concentrated sulfuric acid to evaluate the N content using the Kjeldahl technique. Sodium elements were determined according to the flame photometer using the technique of A.O.A.C. [88]. Silicon content was estimated according to a blue silico molybdate technique, as followed by Novozamsky et al. [89]. The standard curve was set based on SiO_2_ salt. Grain samples were dehydrated and transformed into ash by burning for 3 h at 550 °C. The ash was conveyed to polycarbonate test tubes, and 50 mL of 0.08 M H_2_SO_4_ and 2 mL of 40% hydrogen fluoride were applied. Color development occurred by applying 1.5 mL of this solution to 1.5 mL of reagent mixture (0.08 M H_2_SO_4_ and ammonium molybdate, 20 g L^−1)^; therefore, 1.5 mL of 0.2 M ascorbic acid was applied. The absorbance was measured at 811 nm.

## 5. Conclusions

The findings emphasize the role of plant growth-promoting rhizobacteria (*P. koreensis* and *B. coagulans*) and silicon nanoparticles (nano-Si) in regulating salinity responses and confirm that PGPRs and nano-Si could protect rice plants versus the dangerous impact of soil salinity. Bacterial inoculation leads to the improvement of soil properties and soil enzymes, which creates a suitable environment for plant growth under saline conditions. Spraying the plant with nanofertilizers improves the enzymatic activity, which reduces the risk of oxidation and improves the physiological characteristics of the plant under salinity conditions in salinity-sensitive plants, which is considered a complement to the role of bacterial pollination in improving plant growth and productivity. There were no significant differences between the applications of PGPRs and nano-Si; both forms were valuable options compared to the untreated plots for all of the measured properties to enhance saline tolerance and rice crop yield. Promising impacts of PGPRs and nano-Si on root enhancement, plant growth, and yield attributes have been ascribed to (1) the help of pigments to augment photosynthetic capacity; (2) increasing of assimilates to protect osmosis; (3) increasing antioxidant enzymes activity by removing oxidative stress; and (4) enhancing physiological processes for osmoregulation in the cells. However, further research trials are needed to confirm the obtained results on a large scale. Using molecular approaches to find out the mechanisms behind their synergistic effect in roots and leaves. 

## Figures and Tables

**Table 1:** Impact of microbial inoculants (MI) and silicon nanoparticles (nano-Si) applications on the activity of urease and dehydrogenase in the soil cultivated with rice plants (cultivars Giza 179 and Giza 177) during 2020 and 2021 growing seasons.

Cultivar	Treatments	Urease (mg N-NH_4_^+^ g^−1^ Soil h^−1^)		Dehydrogenase (mg TPF g^−1^ DM Soil d^−1^)	
		2020	2021	2020	2021
Giza 179	Control	103.3 ± 2.4 de	111.5 ± 3.6 d	53.2 ± 3.1 g	56.8 ± 1.5 f
	MI	155.1 ± 1.8 b	170 ± 2.2 b	104 ± 2.1 c	108.8 ± 1.8 c
	Nano-Si	137.1 ± 3.7 c	147.9 ± 2.6 c	84.2 ± 1.2 d	88.6 ± 1.6 d
	MI + nano-Si	202.8 ± 2.4 a	212 ± 3.5 a	139.6 ± 4.1 a	149 ± 3.3 a
Giza 177	Control	73 ± 1.7 f	81.9 ± 2.1 f	30.9 ± 2 h	46.2 ± 2.4 g
	MI	106.3 ± 3.4 d	110.3 ± 2.7 de	77.2 ± 0.7 e	90.5 ± 1.6 d
	Nano-Si	96.3 ± 2.2 e	104 ± 3.6 e	63.1 ± 0.8 f	76.9 ± 2.6 e
	MI + nano-Si	140.8 ± 2.4 c	146.9 ± 1.6 c	110.7 ± 1.9 b	117 ± 1.4 b
Cultivars		***	**	**	**
Treatments		***	***	***	***
Cultivars X Treatments		***	***	*	***

Means ± standard deviation followed by different letters indicate significant differences between treatments according to Tukey’s HSD Test (*p* < 0.05). ***, **, and * denote significance at *p* < 0.001, <0.01, and <0.05, respectively.

**Table 2:** Impact of microbial inoculants (MI) and silicon nanoparticles (nano-Si) applications on the sodium (Na) and potassium (K) ion content in the leaves of rice plants (cultivars Giza 179 and Giza 177) during 2020 and 2021 growing seasons.

Cultivar	Treatments	Na (mg g^−1^ DW)		K (mg g^−1^ DW)		K/Na (Ratio)	
		2020	2021	2020	2021	2020	2021
Giza 179	Control	2.6 ± 0.1 cd	2.74 ± 0.09 d	0.88 ± 0.15 d	0.93 ± 0.08 d	0.34 ± 0.05 de	0.35 ± 0.02 d
	MI	2.18 ± 0.1 ef	2.18 ± 0.15 f	1.23 ± 0.12 bc	1.34 ± 0.04 c	0.56 ± 0.03 c	0.62 ± 0.06 c
	Nano-Si	1.96 ± 0.11 fg	2.04 ± 0.1 f	1.45 ± 0.06 b	1.56 ± 0.08 b	0.74 ± 0.01 b	0.77 ± 0.07 b
	MI + nano-Si	1.65 ± 0.1 g	1.78 ± 0.04 g	1.77 ± 0.12 a	1.91 ± 0.14 a	1.08 ± 0.11 a	1.07 ± 0.1 a
Giza 177	Control	3.52 ± 0.11 a	3.68 ± 0.1 a	0.22 ± 0.07 f	0.35 ± 0.06 f	0.06 ± 0.02 g	0.1 ± 0.01 f
	MI	3.14 ± 0.32 b	3.27 ± 0.14 b	0.57 ± 0.07 e	0.69 ± 0.09 e	0.18 ± 0.04 fg	0.21 ± 0.02 ef
	Nano-Si	2.93 ± 0.11 bc	3.06 ± 0.09 c	0.81 ± 0.04 de	0.83 ± 0.04 de	0.28 ± 0.02 ef	0.27 ± 0.01 de
	MI + nano-Si	2.34 ± 0.11 de	2.45 ± 0.17 e	1.05 ± 0.1 cd	1.20 ± 0.06 c	0.45 ± 0.03 cd	0.49 ± 0.05 c
Cultivars		*	**	**	**	***	**
Treatments		***	***	***	***	***	***
Cultivars X Treatments		ns	***	ns	ns	***	***

Means ± standard deviation followed by different letters indicate significant differences between treatments according to Tukey’s HSD Test (*p* < 0.05). ***, **, and * denote significance at *p* < 0.001, <0.01, and <0.05, respectively.

**Table 3:** Impact of microbial inoculants (MI) and silicon nanoparticles (Si-NPs) applications on nitrogen (N), phosphorus (P), potassium (K), sodium (Na) and silicon (SI) ions content in the grains of rice plants (cultivars Giza 179 and Giza 177) during 2020 and 2021 growing seasons.

Season	Cultivar	Treatments	N (g kg^−1^ DW)	P (g kg^−1^ DW)	K (g kg^−1^ DW)	Na (g kg^−1^ DW)	Si (g kg^−1^ DW)
2020	Giza 179	Control	1.30 ± 0.04 ce	0.41 ± 0.04 cd	0.51 ± 0.05 e	0.44 ± 0.04 cd	0.33 ± 0.02 d
		MI	1.46 ± 0.08 ac	0.54 ± 0.03 cd	0.81 ± 0.04 c	0.32 ± 0.04 de	0.48 ± 0.04 bc
		Si-NPs	1.49 ± 0.07 ab	0.77 ± 0.06 b	1.05 ± 0.08 b	0.24 ± 0.02 e	0.51 ± 0.04 b
		MI + Si-NPs	1.53 ± 0.04 a	1.01 ± 0.09 a	1.33 ± 0.08 a	0.19 ± 0.01 e	0.58 ± 0.03 a
	Giza 177	Control	1.18 ± 0.07 e	0.15 ± 0.06 e	0.29 ± 0.06 f	0.79 ± 0.05 a	0.22 ± 0.04 e
		MI	1.27 ± 0.05 de	0.39 ± 0.02 d	0.55 ± 0.03 de	0.66 ± 0.03 ab	0.33 ± 0.01 d
		Si-NPs	1.32 ± 0.07 ce	0.41 ± 0.03 cd	0.63 ± 0.02 de	0.59 ± 0.01 b	0.44 ± 0.01 c
		MI + Si-NPs	1.35 ± 0.04 bd	0.56 ± 0.02 c	0.72 ± 0.06 cd	0.53 ± 0.03 bc	0.46 ± 0.03 bc
	Cultivars		*	***	**	**	**
	Treatments		***	***	***	***	***
	Cultivars X Treatments		ns	**	***	*	*
2021	Giza 179	Control	1.29 ± 0.06 bc	0.38 ± 0.03 d	0.54 ± 0.05 de	0.46 ± 0.02 d	0.35 ± 0.02 d
		MI	1.45 ± 0.08 ab	0.51 ± 0.04 c	0.88 ± 0.04 c	0.28 ± 0.01 e	0.47 ± 0.02 c
		Si-NPs	1.48 ± 0.08 ab	0.89 ± 0.03 b	1.14 ± 0.08 b	0.22 ± 0.02 e	0.52 ± 0.03 b
		MI + Si-NPs	1.56 ± 0.07 a	1.08 ± 0.05 a	1.44 ± 0.08 a	0.20 ± 0.03 e	0.59 ± 0.04 a
	Giza 177	Control	1.21 ± 0.07 c	0.15 ± 0.02 e	0.38 ± 0.06 e	0.79 ± 0.03 a	0.26 ± 0.02 e
		MI	1.30 ± 0.06 bc	0.35 ± 0.05 d	0.64 ± 0.03 d	0.63 ± 0.01 b	0.35 ± 0.01 d
		Si-NPs	1.32 ± 0.07 bc	0.41 ± 0.04 d	0.67 ± 0.02 cd	0.58 ± 0.01 bc	0.45 ± 0.01 c
		MI + Si-NPs	1.35 ± 0.05 ac	0.55 ± 0.04 c	0.74 ± 0.06 cd	0.51 ± 0.02 cd	0.47 ± 0.02 c
	Cultivars		**	***	***	***	**
	Treatments		**	***	***	***	***
	Cultivars X Treatments		ns	***	***	*	*

Means ± standard deviation followed by different letters indicate significant differences between treatments according to Tukey’s HSD Test (*p* < 0.05). ***, **, and * denote significance at *p* < 0.001, <0.01, and <0.05, respectively.

**Table 4:** Soil physicochemical attributes used in the two summer 2020 and 2021 seasons.

				Soluble Cations (meq L^−1^)				Soluble Anions (meq L^−1^)		
Season	O.M (%)	EC (dS m^−1^)	pH	Na^+^	K^+^	Mg^2+^	Ca^2+^	Cl^−^	HCO_3_^−^	SO_4_^−^
2020	1.30	7.36	8.22	64.14	0.42	8.36	10.14	59.56	8.34	7.45
2021	1.32	7.24	8.12	65.03	0.40	7.24	9.98	60.32	9.26	6.54

O.M = organic matter, EC = electrical conductivity.

## Data Availability

Not applicable.
